# Professional Tennis Players and their subsceptibility for Obsessive-Compulsive and Depressive Symptoms

**DOI:** 10.1192/j.eurpsy.2023.1958

**Published:** 2023-07-19

**Authors:** G. Cappellato

**Affiliations:** 1 University of Pisa, costa di Rovigo, Italy

## Abstract

**Introduction:**

A moderate sport activity is considered beneficial for both physical and mental health. On the contrary, different studies have shown that professional players may be more vulnerable to suffer from psychological and/or psychiatric disorders.

**Objectives:**

Given the limited information available, the present study aimed to investigate the possible presence of depressive and obsessive-compulsive symptoms or disorders in a group of professional tennis players.

**Methods:**

Twenty-five current or former professional tennis players (18 men and 7 women; mean age ± SD: 42.32 ± 13.45 years), were recruited within the Italian Tennis Federation during an international competition and during a master meeting of coaches. They were compared with a control group, recruited from university students, doctors and nurses. All of them underwent a psychiatric interview with a structured scale and a psychopathological assessment carried out with the Mini-International Neuropsychiatric Interview (MINI), the Yale-Brown Obsessive Compulsive Scale (Y-BOCS) and the Self Assessment Scale for Depression (SAD).

**Results:**

The Y-BOCS total and subscale scores were significantly higher in both current and past athletes than controls. Current athletes showed higher scores at Y-BOCS total, subscales and some items. The majority of the current athletes also showed superstitions and magical thinking.

**Conclusions:**

The present study demonstrated that professional tennis players show a relevant increase of obsessive-compulsive symptoms and supertistions than controls. Interestingly, current athletes resulted more severe than past ones. Taken together, our findings support the notion that agonistic sport activities of high level require intensive training and compliance to strict daily routines that might represent a sort of vulnerability toward the onset of full-blown obsessive-compulsive disorder (as well as other disorders) in more fragile individuals. Not suprisingly, sport psychological support experts are increasingly needed.

**Disclosure of Interest:**

None Declared

